# Psychological assessment in infertility: A systematic review and meta-analysis

**DOI:** 10.3389/fpsyg.2022.961722

**Published:** 2022-10-28

**Authors:** Sayed Abolfazl Tavousi, Mohaddeseh Behjati, Alireza Milajerdi, Amir Hossein Mohammadi

**Affiliations:** ^1^Student Research Committee, Kashan University of Medical Sciences, Kashan, Iran; ^2^Cellular, Molecular and Genetics Research Center, Isfahan University of Medical Sciences, Isfahan, Iran; ^3^Research Center for Biochemistry and Nutrition in Metabolic Diseases, Institute for Basic Sciences, Kashan University of Medical Sciences, Kashan, Iran

**Keywords:** infertility, psychological assessment, SCL90, BSI, DASS, STAI

## Abstract

Infertility is a prevalent worldwide health issue and is defined by the World Health Organization (WHO) as a global health problem. Considering the importance of the psychological dimensions of infertility, various measurement tools have been used to measure the variables involved in infertility, of which the most widely used are the following: the Symptom Checklist 90 (SCL90), the Brief Symptom Inventory (BSI), the State-Trait Anxiety Inventory Form (STAI), and the Depression Anxiety Stress Scale (DASS). Therefore, given the problems of infertile people in terms of psychological dimensions, the aim of this meta-analysis was to assess the psychological assessment score in infertility. Following the Preferred Reporting Items for Systematic Reviews and Meta-Analyses (PRISMA) protocol, we applied an online database with no time restriction. Data were gathered using a random-effect model to estimate the standard mean difference (SMD) for the evaluation of the strength of association analyses. Our data demonstrated a significant higher SCL90 score (*CI*_*SCL*90_: 0.96, 0.34–1.57, heterogeneity: 94%, *p*_*heterogeneity*_ < 0.001), and a non-significant higher DASS score (*CI*_*Anxiety*_: 0.82, -0.14 to 1.79; CI_*Depression*_: 0.8, -0.28 to 1.87; and *CI*_*Stress*_: 0.82, -0.24 to 1.88). It is essential to seek for strategies to help infertile patients overcome their infertility-related psychological problems.

## Introduction

Infertility is a widespread, serious health problem that is defined as a global public health problem by the World Health Organization (WHO) (Agarwal et al., 2015; Cong et al., 2016). Infertility is considered as the failed achievement of a viable pregnancy following 1 year of unprotected intercourse, and it is estimated that more than 186 million people suffer from it worldwide (Inhorn and Patrizio, 2015). This period for women over 35 years of age, however, has been considered 6 months (Practice Committee of the American Society for Reproductive Medicine, 2013). The overall estimated burden of subfertility/infertility is announced to be high. Based on the WHO reports, infertility affects over 10% of women (Boivin et al., 2011; Kalkhoran et al., 2011; Direkvand Moghaddam et al., 2016; Sarkar and Gupta, 2016) and 7% of men (Krausz and Riera-Escamilla, 2018). Indeed, the prevalence of secondary infertility is reported to be about 4.9% (Akhondi et al., 2019).

The reasons for infertility might be categorized into four major groups: (1) male factors, (2) female factors, (3) both male and female factors, and (4) unknown etiology (Speroff and Fritz, 2005; Blundell, 2007; Krausz, 2011). Currently, three major therapeutic strategies, such as surgical therapy (especially endoscopic techniques), pharmacological therapy, and assisted reproductive technology (ART), are available to treat male infertility. ART encompasses all interventional measures, including *in vitro* handling of both human sperm and oocytes and of embryos for reproduction issues. ART includes *in vitro* fertilization (IVF) and embryo transfer, gamete and embryo cryopreservation, embryo biopsy, intracytoplasmic sperm injection (ICSI), and preimplantation genetic testing (Dyer et al., 2016).

**FIGURE 1:**
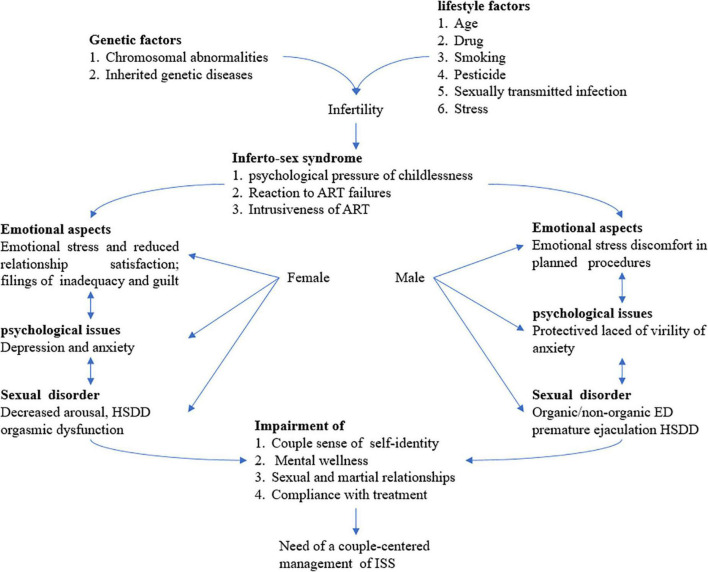
Infertility is a real challenge as a couple disorder and sexual dysfunction is an emerging paradigm. A couple-centered approach for the treatment for infertility is mandatory requiring a multidisciplinary team for the comprehensive management of ISS including the whole sexual, rational, and emotional aspects of infertile couples. ART assisted reproductive technology, ED erectile dysfunction, HSDD hypoactive sexual desire disorder, and ISS Inferto-Sex Syndrome. This figure is adapted from Luca et al. (2021).

Infertility affects different life aspects, such as social, mental, and physical ones (Tanha et al., 2014). It also can lead to the development of stigma, shame, depression, anxiety, guilt (Figure 1), and low self-esteem (2019). It is one of the personal and social issues affecting the whole couple’s life and family function, leading to the development of psychological stress and or psychiatric disorders (Boivin, 2003). Infertility can also be considered as one of the most stressful life events. Infertility is believed to be associated with health issues, feeling of grievance, stressful experiences, depression, lack of self-confidence, disappointment, threat, sin, and marital problems (Noorbala et al., 2008). Infertility can generate stress in the family (Dyer et al., 2005; Ozkan and Baysal, 2006; Sadeghian et al., 2006; Peyvandi et al., 2011; Xu et al., 2017). It is speculated that psychological factors rather than biological ones might be the primary cause of infertility. This can be an important field of interest for psychologists (Podolska, 2011).

Due to the importance of the psychological dimension of infertility, various psychological treatments for infertility have been used so far (Domar et al., 1992; McQueeney et al., 1997; Hosaka et al., 2002; McNaughton-Cassill et al., 2002; Cousineau et al., 2008; Faramarzi et al., 2008; Lancastle and Boivin, 2008; Panagopoulou et al., 2009; Hämmerli et al., 2010; Hughes and da Silva, 2011; Vizheh et al., 2013; Frederiksen et al., 2015). Due to the importance of the psychological dimensions of infertility, various measurement tools have been used to assess the variables involved in infertility. The most widely used factors are the following: the Symptom Checklist 90 (SCL90) (Derogatis et al., 1977), the Brief Symptom Inventory (BSI) (Derogatis and Spencer, 1993), the Depression Anxiety Stress Scale (DASS) (Parkitny and McAuley, 2010a), and the State-Trait Anxiety Inventory Form (STAI) (Spielberger, 1983).

Given the importance of psychological issues and problems in the quality of life and the quality of physical therapy for infertile people, paying attention to the psychological dimensions of this problem is strongly required. On the other hand, due to the wide spectrum of mental problems, and in other words, the subjectivity of psychological problems, it is necessary to be able to measure the severity of psychological problems with small tools and break them down into smaller components so that we can study them in the form of scientific problems. One of the important tools to achieve this goal is the use of self-reported psychometric tools, which, despite the problems that this type of measurement has, can partially explain the total dimensions of the problem. Despite the number of studies investigating the association between psychological assessment scores and infertility, there are still some contradictory results. Thus, we aimed to evaluate the association between the psychological assessment score and infertility patients through a meta-analysis approach.

## Materials and methods

### Search strategy

This study has been done according to the PRISMA protocol (Moher et al., 2009). A systematic search was performed by two independent researchers (SAT and AHM) from the online database to find relevant publications until September 2021. The keywords used in our search strategy were ((”Infertility”) AND (”Psychological assessment” OR “Symptom Checklist-90” OR “SCL90” OR “Brief Symptom Inventory” OR “BSI” OR “Depression Anxiety Stress Scale” OR “DASS” OR “State-Trait Anxiety Inventory Form” OR “STAI”)). No restrictions were considered for the time and language of publications.

### Inclusion and exclusion criteria

Inclusion criteria include cases defined according to infertility diagnosis; all studies evaluating the SCL90, BSI, DASS, and STAI scores of patients with infertility; studies that reported the SCL90, BSI, DASS, and STAI scores at baseline through clinical trials on infertile patients vs. healthy controls; and those that reported mean ± standard deviation (SD). In the case of the same dataset with more than one publication, only articles with more complete findings were included in our study. On the other hand, comments, short letters, clinical trials without a healthy control group, communications, reviews, meta-analyses, case reports, and animal studies were excluded from the meta-analysis.

### Data extraction

Two independent researchers performed data extraction and study selection. The reported mean ± SD for the SCL90, BSI, DASS, and STAI scores were extracted from patients compared with the control group. In addition, we extracted the information from each study (Tables 1–5).

**TABLE 1:** SCL90 assessment studies in infertility.

Type of research	Infertility type	Infertility treatment	Number of samples	Population	Outcome	References
Descriptive	Female	IVF	43	Spain	Significant differences were observed between both groups regarding depression	Santa-Cruz et al. (2020)
Descriptive	Female	IVF	355	Iran	A significant difference in location was observed between two groups. Mental health issues remained stable even after one year since abortion in the RSA group.	Adib-Rad et al. (2019)
Descriptive	Female and male	IVF	256	Iran	Prevalence of depression, paranoia, anxiety and hypochondriasis were significantly higher in infertile females and spouses of infertile males than husbands of infertile females.	Karimzadeh et al. (2017)
Cross-sectional comparative study	Female and male	No treatment	120	India	For the variable impact of event, somatization, interpersonal sensitivity, anger hostility, paranoid ideation, global severity, irrational belief significant difference was found between the genders	De et al. (2017)
Cross sectional	Female	IVF	470	Swedish	Women with previous history of IVF treatment were at increased risk of symptoms of depression, somatization and obsessive-compulsion compared to a reference group.	Vikström et al. (2015)
Descriptive	Female	IVF	247	Palestine	Statistically significant differences between fertilized wives and infertile wives in the psychopathological symptoms in favor of infertile wives.	Hamdouna (2014)
Not mentioned	Female	Not mentioned	150	Iran	Results demonstrated that 28.7% (43 women) of fertile and 44% (66 women) of infertile group had psychological disorders, thus significantly higher prevalence of psychological disorders was observed among infertile vs. fertile women.	Abedinia et al. (2013)
Descriptive	Female	IVF	100	China	Worse mental status of infertile women under treatment with IVF-ET was observed compared to fertile women.	Chen et al. (2008)
Descriptive	Female	IVF	300	China	Significantly higher scores on all subscales of the SCL-90 was observed in infertile Chinese women who planned to undergo IVF-ICSI.	Wang et al. (2007)
Descriptive- analytic study	Female	Not mentioned	300	Iran	The highest mean scores were observed for depression, paranoid ideation, and interpersonal sensitivity scales and the lowest scores were attributed to psychoticism and phobic anxiety scales among infertile women. Interpersonal sensitivity, phobic anxiety, depression, paranoid ideas and psychoticism scales were significantly different between fertile and infertile women.	Noorbala et al. (2007)
Descriptive	Female	Not mentioned	120	South Africa	Significantly higher scores on all sub-scales and global distress indices of the SCL-90-R vs. controls.	Dyer et al. (2005)

**TABLE 2:** BSI assessment studies in infertility.

Type of research	Infertility type	Infertility treatment	Number of samples	Population	Outcome	References
Retrospective, descriptive and causal study	Female and men	IVF	278	Portugal	Somatization, depression, anxiety, phobic anxiety, as well as Global severity index (GSI), total of positive symptoms (TPS) and positive symptoms index (PSI) are always significantly higher for women	Pereira et al. (2020)
Prospective, study with consecutive	Female	cIVF or NC-IVF	119	Switzerland	Baseline significantly more overall psychological distress BSI GSI and signs of obsession. Infertile women undergoing c IVF treatment displayed a significantly higher level of depression after the treatment in comparison with the NC-IVF patients	Haemmerli Keller et al. (2018)
Descriptive	Female	IVF	203	Germany	Infertile women and men with reduced levels of depression presented stronger intentions to continue. The anxiety and the somatization didn’t evidence any significant impact in couples’ intention to persist in treatments.	Marques et al. (2017)
Correlational methodology	Female	IVF/ICSI	79	Iran	the variables of stress, depression, overall severity index, positive symptom distress index, interpersonal sensitivity, and obsessive-compulsive disorder predict this likelihood that the IVF / ICSI treatments will be successful	Aslzaker et al. (2016)
Exploratory study	Female and men	ART	433	Portugal	Presents direct and indirect associations found between PCC and at least one measure of wellbeing presents direct, indirect and total effects of PCC on depression. Direct effects were found for communication, respect for patients’ values.	Gameiro et al. (2013)
Exploratory study	Female and men	IVF	383	Portugal	Anxiety and depression, as measured with the screen IVF, were more strongly related with the BSI subscales of anxiety and depression.	Lopes (2012)
Prospective, longitudinal design	Female	IVF	121	Israel	Pregnant women did not differ from non-pregnant women in any of the psychological symptoms measured at ovulation induction	Zaig et al. (2012)
Exploratory study	Female	IVF	208	Belgium	There were no clinically significant differences with the published norms	Van den Broeck et al. (2010)

**TABLE 3:** DASS assessment studies in infertility.

Type of research	Infertility type	Infertility treatment	Number of samples	Population	Outcome	References
Therapy	Female	IVF	60	Iran	Mean score of The post-intervention of depression and stress was significantly lower in the intervention vs. control group.	Rahimi et al. (2021)
Therapy	Female	IVF	144	Greece	The difference of stress levels between the two groups for each scale as well as in total was also significant	Koumparou et al. (2021)
Cross-sectional study	Female	polycystic ovary syndrome	120	Iran	Stress, depression, and anxiety total scores a significant negative correlation was found between each dimension of DASS scale.	Naz et al. (2020)
Case control study	Female		200	Pakistan	Depression, stress and anxiety were highly prevalent among infertile females vs. control group.	Yusuf (2016)
Correlational methodology	Female	IVF/ICSI	79	Iran	Predictors of anxiety, depression, and stress, quality of life, brief symptom, infertility stress and infertilityhe, all of them can explain 25% of the variance of the dependent variable – ICSI / IVF treatment success.	Aslzaker et al. (2016)
Prospective cohort study	Female	IUI/IVF	206	Korea	There was a significantly higher scores of anxiety, depression and stress among infertile vs. fertile women.	Chi et al. (2016)
Cross-sectional study	Female	Polycystic ovary syndrome	16	Malaysia	Patients with symptoms of anxiety and depression were more likely to suffer from sexual dysfunction than other. Higher orgasm dysfunction was also observed in cases with stress symptoms than other.	Dashti et al. (2016)
Psycho-education	Female	IVF	64	Macedonia	Psycho-education resulted in getting lower anxiety and stress scores, while depression scales did not show differences between groups.	Belevska (2015)
Pilot study	Female and men	IVF/ICSI	246	Malaysia	Depression, stress and anxiety-related difficulties were significantly higher in wives than their husbands	Musa et al. (2014)

**TABLE 4:** STAI-S assessment studies in infertility.

Type of research	Infertility type	Infertility treatment	Number of samples	Population	Outcome	References
Descriptive, correlational study	Female	No treatment	240	Romania	Higher scores on the anxiety obtained at the women who were beginning of treatment.	Iordãchescu et al. (2021)
Prospective cohort study	Female	IVF	304	Kazakhstan	Anxiety scores were significantly lower among pregnant women than not pregnant women.	Aimagambetova et al. (2020)
Cross-sectional study	Female	IVF	161	Spain	Higher anxiety-state were observed but, no significant differences in depression scores were found among both groups.	Lasheras et al. (2020)
Pilot study	Female	IVF	44	United States	At all three time points, mean STAI State scores were significantly elevated.	Turner et al. (2013)
Prospective study.	Female	IVF	24	Brazil	No significant differences among the average values obtained during these 4 phases of major anxiety in women treated with IVF.	Seibel et al. (2003)

**TABLE 5:** General characteristics of included studies in the meta-analysis.

Country	Case		Control		Treatment	Sample size (Ca/Co)	Study design	Age		Gender	Match	Quality assessment	Result	References
	Mean	SD	Mean	SD				Ca	Co					
Iran	11.67	16.9	10.63	9.63	IVF	(128/128)	Descriptive analytical	NT	NT	F and M	NT	4	↑	Karimzadeh et al. (2017)
India	1.53	0.29	1.02	0.02	IVF	(20/30)	Cross-sectional comparative study	NT	NT	F and M	F and M factor	5	↑	De et al. (2017)
China	53.16	4.14	42.1	32.3	IVF	(285/98)	Descriptive	NT	NT	F	Infertility type	4	↑	Wang et al. (2007)
South Africa	65.9	6	59.3	9.5	NT	(120/120)	Descriptive	29.2	28	F	Levels of care	5	↑	Dyer et al. (2005)
Iran	NA	NA	NA	NA	IVF	(26/26)	Randomized controlled trial	34.4 ± 6	32.9 ± 7.4	F	NT	4 (J)	↓	Rahimi et al. (2021)
Pakistan	NA	NA	NA	NA	IVF	(100/100)	Case control study	NT	NT	F	NT	5	↑	Yusuf (2016)
Korea	NA	NA	NA	NA	IVF	(141/65)	Prospective cohort study	34.3 ± 3.7	35.4 ± 4.6	F	Age	6	↑	Chi et al. (2016)
Kazakhstan	49.9	7.2	45.1	9.9	IVF	(48/181)	Prospective cohort study	34.8 ± 5.6	33.8 ± 6.2	F	NT	5	↑	Aimagambetova et al. (2020)
Israel	5.4	2.32	4.99	2.23	IVF	(21/87)	Prospective-longitudinal	32.71 ± 5.1	31.51 ± 5.5	F	NT	5	=	Zaig et al. (2012)

Ca, case; Co, control; F, female; M, male; NT, not mention; NA, not applicable; J-JADAD checklist.

### Quality assessment

A form of the Newcastle Ottawa scale (NOS) was designed for observational studies and was used to assess the quality of selected studies. The NOS considers a maximum of ten points for each study. The studies that had an NOS score of 5 or more were considered high quality publications (Peterson et al., 2011). We used the JADAD checklist for evaluation of the quality of interventional studies (Jadad et al., 1996). Two independent reviewers filled out the scores for each eligible study.

### Statistical analysis

For both the infertility and control groups, the SCL90, BSI, DASS, and STAI scores were reported using mean ± SD and 95% confidence interval (*CI*). The overall mean ± SD data were calculated using a random-effect model and/or a fixed-effect model. The SMD and *CI* were considered the overall if there was true heterogeneity between included studies, we employed a random-effect model; otherwise, a fixed-effect model was used. To assess between-study heterogeneity, Cochran’s *Q*-test and *I*^2^ were used. A random-effect model was used if *p*_*heterogeneity*_ was less than 0.1 (Higgins et al., 2011). Statistical analyses were carried out using review manager software (STATA version 14, StataCorp).

## Result

**FIGURE 2:**
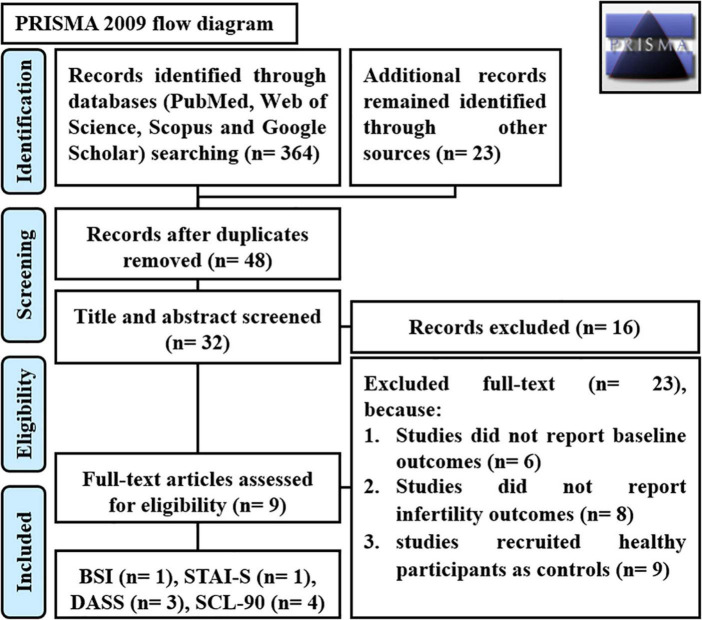
PRISMA flow diagram. SCL90 Symptom Checklist 90, BSI Brief Symptom Inventory, STAI State-Trait Anxiety Inventory Form, and DASS Depression Anxiety Stress Scale.

**FIGURE 3:**
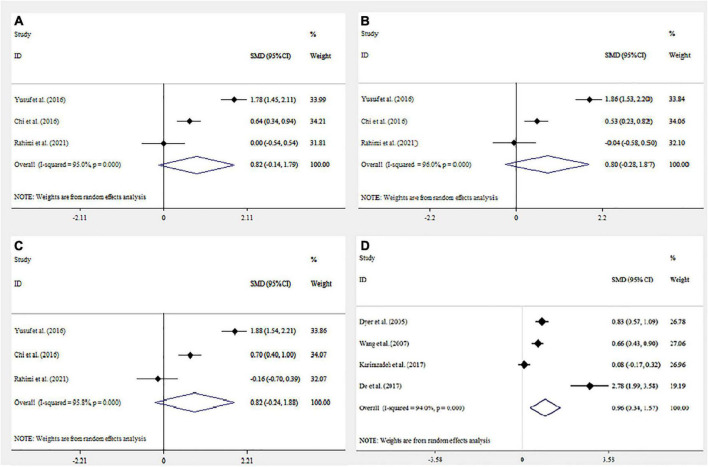
Forest plot mean difference and 95% confidence intervals (CI) of the meta-analysis on psychological assessment score and association with infertility: Depression Anxiety Stress Scale (DASS) **(A–C)** (Anxiety, Depression, and Stress respectively); Symptom Checklist 90 (SCL90) **(D)**. Horizontal lines represent 95% CI.

We identified 32 studies in our systematic review. The last remaining nine articles were included in the meta-analysis, and the characteristics of these studies are illustrated in Figure 2. The results of the overall and stratified analyses are summarized in Figure 3. Regarding SCL90 and DASS, there was a statistically significant association between higher SCL90 scores and infertility. However, there was no significant difference between the case and control groups regarding the higher score of DASS. Indeed, true heterogeneities were observed across studies for the two abovementioned parameters.

## Discussion

In our meta-analysis, we found a statistically significant higher score of SCL90 but there was not a statistically significant association between a higher score of DASS and infertility. Only one study was performed regarding the relationship between BSI and STAI in association with infertility. Therefore, further study is needed to investigate the association between infertility and BSI and STAI. In line with our findings, Petersen et al. in gynecologic cancer (Petersen et al., 2005), Shi et al. in polycystic ovarian syndrome (PCOS) (Shi et al., 2011), and Pan et al. (2013) in breast cancer reported higher SCL90 scores in subjects compared with the control group. Similar investigations were also performed. Barber and Steadman demonstrated a non-significantly different DASS score in pregnant vs. non-pregnant women (Barber and Steadman, 2018). In contrast, Rothemund et al. observed no significant difference regarding the SCL90 score in women at high risk for breast cancer (Rothemund et al., 2001). Furthermore, Ali et al., Jamshidian-QalehShahi et al., and Ho et al. reported higher DASS scores in subjects with infertility (Ali et al., 2011; Jamshidian-QalehShahi et al., 2017; Ho et al., 2020).

In comparison with the DASS, the SCL90 is used to measure more general psychological symptoms (Eivors et al., 2008). Additionally, it examines more general and complete psychological distress among people compared with the DASS score (Modabernia et al., 2010). Indeed, the physicalization and obsessive-compulsive scales, which are higher in infertile cases, are included in the SCL90 while they are not measured by the DASS score (Conrad et al., 2003; Burns, 2007). One of the scales which is examined more completely in the SCL90 test than the DASS score and is also observed in infertile people is interpersonal sensitivity, i.e., infertile people usually have a higher score on this scale due to the psychological nature of infertility (Noorbala et al., 2009). Furthermore, one of the scales that exists meaningfully in infertile people and is measured by the SCL90 test is paranoid ideations due to the psychological burden of bereavement in infertility (Sadeghian et al., 2006). In general, the SCL90 test examines the psychological symptoms caused by infertility in a wider way than the DASS test, and it is a more reliable scale for measuring the overall symptoms. However, the DASS test, which is applied for the measurement of depression, stress, and anxiety, is more specialized than the SCL90 since it examines these three scales, and if the goal is only to examine these three scales, the DASS test will be more specialized (Parkitny and McAuley, 2010b).

The components of anxiety and depression exist in infertile patients. Since the nature of infertility is felt by infertile people, such as experiencing grief and experiencing a feeling of deficiency in the body, the intensity of these components will increase psychologically in these people. Generally, some aspects of depression are caused by the feeling of impaired efficiency (Lapane et al., 1995). In addition, biological data indicate decreased serotonin and norepinephrine levels in these people following the emergence of depression problems (Shi et al., 2011). Studies demonstrate the negative impacts of these two factors on fertility treatment (Szkodziak et al., 2020).

A study on a rat sample of depression and menopause illustrated significantly higher levels of follicle-stimulating hormone (FSH), Luteinizing hormone (LH), cortisone, and Adrenocorticotropic hormone (ACTH) in groups exposed to mild stress factors vs. those without such an exposure. On the other hand, dopamine levels were lower in the stress groups (Gu et al., 2018).

Men and women equally suffer from obsessive-compulsive disorder (OCD) that most often develops in early life. Infertile cases with a history of OCD might be focused on contamination obsessions and cleaning rituals associated with infertility treatments due to their assumption regarding sterile techniques associated with injections as a part of various treatments for infertility (Williams and Koran, 1997) that can enhance their exclusive compulsive ritualistic behaviors and or trigger their newly emerged rituals. Treatment compliance can be impaired due to the existing needle and blood injury phobias and their associated unmanageable anxiety state. Caregivers might be required to adapt therapeutic protocols or request psychological or psychiatric care before going on treatment (Williams and Zappert, 2006). Cognitive behavioral therapies and behavioral strategies should also be encouraged (Williams and Zappert, 2006). A close association was observed between hypothalamus-pituitary disorders and psychological scores in PCOS. In the current study, we observed a significant positive correlation between serum concentration of 3-methoxy-4-hydroxyphenylglycol and phobic subscale scores of SCL90 in total samples. Hostility subscale scores demonstrated a significant negative correlation with serum concentration of Dihydroxyphenylacetic acid of PCOS cases, however, no significant correlation was observed between other monoamine neurotransmitters and their metabolites and other subscales of the SCL90 score (Shi et al., 2011).

Several lines of research support the positive impacts of harp therapy, group psychotherapy, cognitive behavioral therapy, and mind-body intervention (Eastern body-mind-spirit, Integrative body-mind-spirit, and mind-body intervention) on the pregnancy rate, anxiety level, or marital function of infertile couples (Ying et al., 2016). A complex intervention should be developed based on sound evidence to target both men and women in infertile couples who have undergone IVF treatment, especially during the stressful interval waiting for the results of pregnancy tests following failed cycles.

This meta-analysis has several limitations. First, the main limitation is the insufficient number of eligible performed studies containing the subgroup analysis. Second, psychological symptoms are non-specific, and they can be affected by various factors. Although some of these factors, such as age and gender, were considered in included studies, not all of them have been considered in the total associated factors. For a better understanding of the association between psychological status and infertility, it is advisable to consider all of the abovementioned factors. Additionally, the included studies derived from different sample sources and diverse applied methodologies, will naturally increase the data heterogeneity. Last, the low number of eligible articles, the moderate quality (mean of 5 in the NOS), and the high heterogeneity of effect sizes among the included studies in each independent meta-analysis are factors that affect reliability and are better avoided. Some other confounding factors, such as disease phase (acute or remission phase), the age of the disease onset, and the presence of concomitant psychopharmacological treatments of patients, were not considered in the meta-analysis.

In conclusion, we found evidence supporting an increased significant SCL90 score and a non-significant higher DASS score in infertility patients. The prevalence of psychological problems in infertile cases seems to be increasing in many countries. These findings suggest that psychological treatment might have some beneficial impacts on infertility cases. Further research is needed to confirm this association.

## Author contributions

SAT, AHM, and AM designed the study. SAT, AHM, and AM collected data. SAT, AHM, AM, and MB wrote the manuscript. All authors read and approved the final manuscript.
